# (*E*)-*N*′-(3,4-Dimethoxy­benzyl­idene)-2-(8-quinol­yloxy)acetohydrazide–methanol–water (1/1/1)

**DOI:** 10.1107/S1600536809051034

**Published:** 2009-11-28

**Authors:** Zhan-Ling Ma

**Affiliations:** a College of Chemistry and Chemical Engineering of Bohai University, Jinzhou, Liaoning 121000, People’s Republic of China

## Abstract

In the title compound, C_20_H_19_N_3_O_4_·CH_4_O·H_2_O, the Schiff base mol­ecule is almost planar, with a dihedral angle of 1.2 (1)° between the benzene ring and the quinoline ring system. An intra­molecular N—H⋯O hydrogen bond generates an *S*(6) ring. In the crystal, the methanol and water solvent mol­ecules are linked to the Schiff base mol­ecule *via* N—H⋯O, O—H⋯O, O—H⋯N and O—H⋯(O,N) hydrogen bonds.

## Related literature

For background to the applications of 8-hydroxy­quinoline and its derivatives, see: Bratzel *et al.* (1972 ▶); Karmakar *et al.* (2007 ▶); Pierre *et al.* (2003 ▶). For a Schiff base compound containing 2,5-dimethoxy­benzaldehyde, see: Wang *et al.* (2009 ▶). For bond-length data, see: Allen *et al.* (1987 ▶).
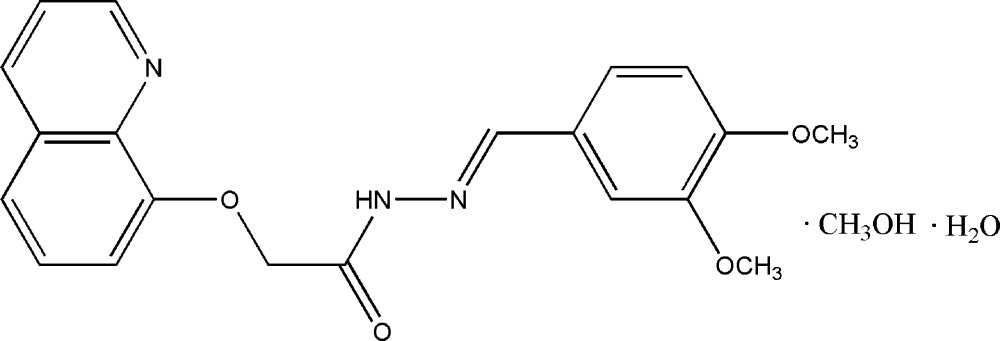


## Experimental

### 

#### Crystal data

C_20_H_19_N_3_O_4_·CH_4_O·H_2_O
*M* *_r_* = 415.44
Triclinic, 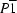

*a* = 8.807 (2) Å
*b* = 10.071 (3) Å
*c* = 13.121 (3) Å
α = 68.702 (4)°
β = 74.552 (3)°
γ = 82.458 (5)°
*V* = 1044.4 (4) Å^3^
*Z* = 2
Mo *K*α radiation
μ = 0.10 mm^−1^
*T* = 295 K
0.21 × 0.18 × 0.16 mm

#### Data collection

Siemens SMART CCD diffractometer
Absorption correction: multi-scan (*SADABS*; Sheldrick, 1996 ▶) *T*_min_ = 0.980, *T*_max_ = 0.985
5612 measured reflections
3676 independent reflections
1571 reflections with *I* > 2σ(*I*)
*R*_int_ = 0.035

#### Refinement

*R*[*F* ^2^ > 2σ(*F* ^2^)] = 0.052
*wR*(*F* ^2^) = 0.126
*S* = 1.01
3676 reflections
273 parameters
H-atom parameters constrained
Δρ_max_ = 0.19 e Å^−3^
Δρ_min_ = −0.17 e Å^−3^

### 

Data collection: *SMART* (Siemens, 1996 ▶); cell refinement: *SAINT* (Siemens, 1996 ▶); data reduction: *SAINT*; program(s) used to solve structure: *SHELXS97* (Sheldrick, 2008 ▶); program(s) used to refine structure: *SHELXL97* (Sheldrick, 2008 ▶); molecular graphics: *SHELXTL* (Sheldrick, 2008 ▶); software used to prepare material for publication: *SHELXTL*.

## Supplementary Material

Crystal structure: contains datablocks global, I. DOI: 10.1107/S1600536809051034/hb5253sup1.cif

Structure factors: contains datablocks I. DOI: 10.1107/S1600536809051034/hb5253Isup2.hkl

Additional supplementary materials:  crystallographic information; 3D view; checkCIF report

## Figures and Tables

**Table 1:** Hydrogen-bond geometry (Å, °)

*D*—H⋯*A*	*D*—H	H⋯*A*	*D*⋯*A*	*D*—H⋯*A*
N2—H8⋯O1	0.86	2.35	2.698 (3)	105
N2—H8⋯O5^i^	0.86	2.06	2.899 (3)	165
O6—H6⋯O2	0.82	1.98	2.756 (4)	156
O6—H6⋯N3	0.82	2.58	3.194 (4)	133
O5—H21⋯N1^ii^	0.85	2.00	2.834 (4)	168
O5—H22⋯O6^iii^	0.85	2.02	2.847 (4)	164

Symmetry codes: (i) 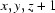; (ii) 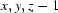; (iii) 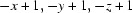.

**Fig. 1.**
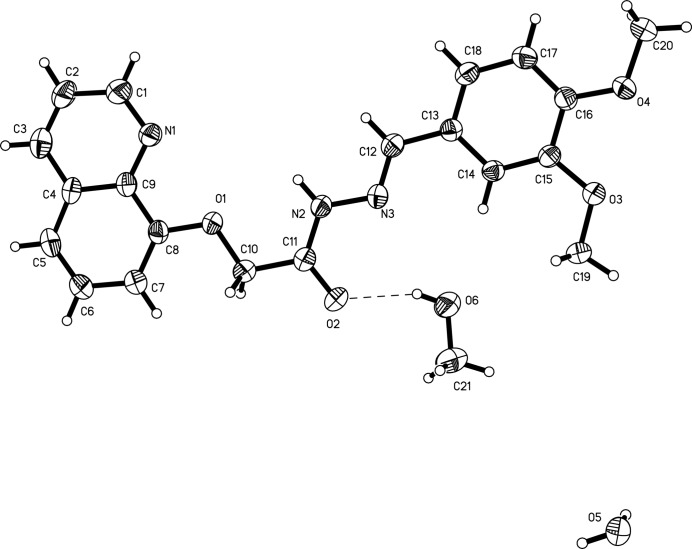
The molecular structure of (I), with displacement ellipsoids drawn at the 30% probability level. The dashed lines indicate hydrogen bonds.

## supplementary crystallographic information

### Comment

8-Hydroxyquinoline and its derivatives have been used widely in analytical chemistry (Bratzel *et al.*, 1972), coordination chemistry (Karmakar *et al.*, 2007), pharmaceutical chemistry (Pierre *et al.*, 2003) and many other topics. As part of our on going search for good extractants of metal ions or a biologically active material, the title compound was obtained in the reaction of quinolin-8-yloxyacetic acid hydrazide and 3,4-dimethoxybenzaldehyde.

The Schiff base molecule of the compound displays a *trans* configuration with respect to the C=N and C—N bonds(Fig. 1). All the bond lengths are within normal(Allen *et al.*, 1987), and are comparable to those in the related compound (*E*)-*N'*-(2,5-Dimethoxybenzylidene)-2-(8- quinolyloxy)acetohydrazide methanol solvate (Wang *et al.*, 2009). The molecule is nearly planar, with a dihedral angle of the benzene ring and the quinoline ring is 1.2 (1)°. The methanol and water solvate molecules are linked to the host *via* N—H···O, O—H···O and O—H···N hydrogen bonds(Table 1).

### Experimental

3,4-Dimethoxybenzaldehyde (0.1 mmol, 16.6 mg) and 2-(quinolin-8-yloxy) acetohydrazide (2.18 g, 10 mmol), were dissolved in a 95% ethanol solution (10 ml). The mixture was stirred at room temperature to give a clear colorless solution. Colourless blocks of (I) were formed by gradual evaporation of the solvent over a period of six days at room temperature.

### Refinement

All H atoms were initially located in a difference Fourier map. C-bound H atoms were constrained to an ideal geometry, with C—H = 0.93–0.97 Å, O—H = 0.82–0.85 Å and N—H = 0.86 Å. *U*_iso_(H) = 1.2*U*_eq_(C,*N*), and 1.5*U*_eq_(O).

### Crystal data

**Table:**

C_20_H_19_N_3_O_4_·CH_4_O·H_2_O	*Z* = 2
*M**_r_* = 415.44	*F*(000) = 440
Triclinic, *P*1	*D*_x_ = 1.321 Mg m^−^^3^
Hall symbol: -P 1	Mo *K*α radiation, λ = 0.71073 Å
*a* = 8.807 (2) Å	Cell parameters from 583 reflections
*b* = 10.071 (3) Å	θ = 2.6–22.5°
*c* = 13.121 (3) Å	µ = 0.10 mm^−^^1^
α = 68.702 (4)°	*T* = 295 K
β = 74.552 (3)°	Block, colourless
γ = 82.458 (5)°	0.21 × 0.18 × 0.16 mm
*V* = 1044.4 (4) Å^3^	

### Data collection

**Table:**

Siemens SMART CCD diffractometer	3676 independent reflections
Radiation source: fine-focus sealed tube	1571 reflections with *I* > 2σ(*I*)
graphite	*R*_int_ = 0.035
φ and ω scans	θ_max_ = 25.1°, θ_min_ = 1.7°
Absorption correction: multi-scan (*SADABS*; Sheldrick, 1996)	*h* = −10→10
*T*_min_ = 0.980, *T*_max_ = 0.985	*k* = −10→11
5612 measured reflections	*l* = −15→15

### Refinement

**Table:**

Refinement on *F*^2^	Secondary atom site location: difference Fourier map
Least-squares matrix: full	Hydrogen site location: inferred from neighbouring sites
*R*[*F*^2^ > 2σ(*F*^2^)] = 0.052	H-atom parameters constrained
*wR*(*F*^2^) = 0.126	*w* = 1/[σ^2^(*F*_o_^2^) + (0.0363*P*)^2^ + 0.0145*P*] where *P* = (*F*_o_^2^ + 2*F*_c_^2^)/3
*S* = 1.00	(Δ/σ)_max_ < 0.001
3676 reflections	Δρ_max_ = 0.19 e Å^−^^3^
273 parameters	Δρ_min_ = −0.16 e Å^−^^3^
0 restraints	Extinction correction: *SHELXL97* (Sheldrick, 2008), Fc^*^=kFc[1+0.001xFc^2^λ^3^/sin(2θ)]^-1/4^
Primary atom site location: structure-invariant direct methods	Extinction coefficient: 0.0063 (11)

### Special details

**Table:**

Geometry. All e.s.d.'s (except the e.s.d. in the dihedral angle between two l.s. planes) are estimated using the full covariance matrix. The cell e.s.d.'s are taken into account individually in the estimation of e.s.d.'s in distances, angles and torsion angles; correlations between e.s.d.'s in cell parameters are only used when they are defined by crystal symmetry. An approximate (isotropic) treatment of cell e.s.d.'s is used for estimating e.s.d.'s involving l.s. planes.
Refinement. Refinement of *F*^2^ against ALL reflections. The weighted *R*-factor *wR* and goodness of fit *S* are based on *F*^2^, conventional *R*-factors *R* are based on *F*, with *F* set to zero for negative *F*^2^. The threshold expression of *F*^2^ > σ(*F*^2^) is used only for calculating *R*-factors(gt) *etc*. and is not relevant to the choice of reflections for refinement. *R*-factors based on *F*^2^ are statistically about twice as large as those based on *F*, and *R*- factors based on ALL data will be even larger.

### Fractional atomic coordinates and isotropic or equivalent isotropic displacement parameters (Å^2^)

**Table:**

	*x*	*y*	*z*	*U*_iso_*/*U*_eq_	
O1	0.9264 (2)	0.2984 (2)	1.08007 (16)	0.0553 (6)	
O2	0.7983 (3)	0.3641 (2)	0.83136 (18)	0.0739 (7)	
O3	0.3497 (2)	0.9140 (2)	0.60039 (16)	0.0594 (6)	
O4	0.2433 (3)	1.1261 (2)	0.66550 (17)	0.0672 (7)	
O5	0.7438 (3)	0.5521 (2)	0.15037 (15)	0.0700 (7)	
H21	0.7970	0.4803	0.1834	0.105*	
H22	0.6479	0.5351	0.1841	0.105*	
O6	0.5689 (3)	0.4737 (3)	0.7121 (2)	0.0931 (9)	
H6	0.6306	0.4628	0.7519	0.140*	
N1	0.9468 (3)	0.3418 (3)	1.2638 (2)	0.0573 (8)	
N2	0.7469 (3)	0.4881 (3)	0.95154 (19)	0.0507 (7)	
H8	0.7600	0.4950	1.0121	0.061*	
N3	0.6533 (3)	0.5866 (3)	0.88784 (19)	0.0517 (7)	
C1	0.9595 (4)	0.3630 (4)	1.3543 (3)	0.0787 (12)	
H1	0.9108	0.4449	1.3675	0.094*	
C2	1.0419 (5)	0.2701 (4)	1.4323 (3)	0.0831 (12)	
H2	1.0470	0.2908	1.4948	0.100*	
C3	1.1133 (4)	0.1508 (4)	1.4153 (3)	0.0739 (11)	
H3	1.1686	0.0883	1.4660	0.089*	
C4	1.1039 (4)	0.1214 (4)	1.3206 (3)	0.0542 (9)	
C5	1.1758 (4)	−0.0007 (4)	1.2967 (3)	0.0676 (10)	
H5	1.2304	−0.0675	1.3459	0.081*	
C6	1.1660 (4)	−0.0212 (4)	1.2027 (3)	0.0737 (11)	
H6A	1.2138	−0.1023	1.1878	0.088*	
C7	1.0845 (4)	0.0785 (4)	1.1268 (3)	0.0627 (10)	
H7	1.0804	0.0636	1.0617	0.075*	
C8	1.0114 (4)	0.1970 (3)	1.1484 (3)	0.0506 (8)	
C9	1.0192 (3)	0.2217 (3)	1.2462 (2)	0.0487 (8)	
C10	0.9210 (4)	0.2751 (3)	0.9812 (2)	0.0556 (9)	
H10A	0.8837	0.1802	1.0017	0.067*	
H10B	1.0269	0.2788	0.9338	0.067*	
C11	0.8163 (4)	0.3817 (4)	0.9153 (3)	0.0541 (9)	
C12	0.5916 (4)	0.6888 (3)	0.9221 (2)	0.0531 (9)	
H12	0.6081	0.6919	0.9883	0.064*	
C13	0.4960 (4)	0.8004 (3)	0.8595 (2)	0.0481 (8)	
C14	0.4684 (3)	0.7989 (3)	0.7596 (2)	0.0469 (8)	
H14	0.5083	0.7229	0.7348	0.056*	
C15	0.3838 (4)	0.9073 (3)	0.6981 (2)	0.0486 (8)	
C16	0.3235 (4)	1.0225 (3)	0.7344 (3)	0.0520 (9)	
C17	0.3500 (4)	1.0262 (3)	0.8314 (3)	0.0613 (10)	
H17	0.3111	1.1031	0.8553	0.074*	
C18	0.4354 (4)	0.9145 (4)	0.8945 (3)	0.0590 (9)	
H18	0.4517	0.9170	0.9610	0.071*	
C19	0.4215 (4)	0.8044 (3)	0.5551 (2)	0.0688 (11)	
H19A	0.5339	0.8039	0.5433	0.103*	
H19B	0.3950	0.8226	0.4847	0.103*	
H19C	0.3836	0.7134	0.6071	0.103*	
C20	0.1775 (5)	1.2441 (4)	0.6990 (3)	0.0983 (14)	
H20A	0.0973	1.2123	0.7675	0.147*	
H20B	0.1319	1.3124	0.6411	0.147*	
H20C	0.2586	1.2877	0.7111	0.147*	
C21	0.6316 (5)	0.4092 (5)	0.6337 (3)	0.1262 (19)	
H21A	0.7383	0.4372	0.5979	0.189*	
H21B	0.5703	0.4378	0.5781	0.189*	
H21C	0.6301	0.3076	0.6700	0.189*	

### Atomic displacement parameters (Å^2^)

**Table:**

	*U*^11^	*U*^22^	*U*^33^	*U*^12^	*U*^13^	*U*^23^
O1	0.0614 (15)	0.0611 (15)	0.0474 (12)	0.0089 (12)	−0.0221 (12)	−0.0206 (11)
O2	0.092 (2)	0.0846 (18)	0.0659 (15)	0.0131 (14)	−0.0410 (14)	−0.0398 (14)
O3	0.0733 (17)	0.0556 (14)	0.0531 (14)	0.0098 (12)	−0.0224 (12)	−0.0219 (11)
O4	0.0853 (18)	0.0579 (15)	0.0612 (14)	0.0245 (13)	−0.0244 (13)	−0.0280 (12)
O5	0.0792 (17)	0.0696 (16)	0.0639 (14)	0.0069 (13)	−0.0289 (13)	−0.0207 (12)
O6	0.089 (2)	0.124 (2)	0.095 (2)	0.0277 (17)	−0.0455 (16)	−0.0652 (18)
N1	0.065 (2)	0.0573 (19)	0.0494 (17)	0.0004 (15)	−0.0156 (15)	−0.0175 (14)
N2	0.0532 (18)	0.0560 (18)	0.0441 (15)	0.0010 (15)	−0.0155 (14)	−0.0167 (14)
N3	0.0538 (18)	0.0518 (17)	0.0472 (16)	−0.0010 (15)	−0.0170 (14)	−0.0110 (14)
C1	0.100 (3)	0.078 (3)	0.067 (2)	0.013 (2)	−0.032 (2)	−0.032 (2)
C2	0.109 (3)	0.093 (3)	0.056 (2)	0.006 (3)	−0.041 (2)	−0.024 (2)
C3	0.072 (3)	0.075 (3)	0.069 (3)	−0.006 (2)	−0.032 (2)	−0.008 (2)
C4	0.048 (2)	0.061 (2)	0.048 (2)	−0.0069 (19)	−0.0186 (18)	−0.0063 (18)
C5	0.057 (2)	0.062 (3)	0.078 (3)	0.007 (2)	−0.028 (2)	−0.011 (2)
C6	0.076 (3)	0.064 (3)	0.087 (3)	0.018 (2)	−0.032 (2)	−0.032 (2)
C7	0.062 (2)	0.062 (2)	0.066 (2)	0.007 (2)	−0.022 (2)	−0.023 (2)
C8	0.043 (2)	0.058 (2)	0.049 (2)	−0.0002 (18)	−0.0127 (17)	−0.0151 (18)
C9	0.039 (2)	0.052 (2)	0.049 (2)	−0.0065 (17)	−0.0088 (17)	−0.0100 (17)
C10	0.057 (2)	0.059 (2)	0.057 (2)	0.0000 (18)	−0.0172 (18)	−0.0246 (17)
C11	0.055 (2)	0.058 (2)	0.050 (2)	−0.0028 (19)	−0.0158 (18)	−0.0168 (18)
C12	0.056 (2)	0.056 (2)	0.047 (2)	−0.0072 (19)	−0.0125 (18)	−0.0154 (18)
C13	0.050 (2)	0.048 (2)	0.0437 (19)	−0.0044 (17)	−0.0084 (17)	−0.0136 (16)
C14	0.051 (2)	0.045 (2)	0.0432 (19)	−0.0037 (17)	−0.0080 (17)	−0.0161 (16)
C15	0.050 (2)	0.053 (2)	0.0426 (19)	−0.0046 (17)	−0.0089 (17)	−0.0161 (17)
C16	0.053 (2)	0.052 (2)	0.046 (2)	0.0033 (18)	−0.0085 (17)	−0.0154 (17)
C17	0.068 (3)	0.058 (2)	0.059 (2)	0.006 (2)	−0.013 (2)	−0.0263 (19)
C18	0.069 (3)	0.065 (2)	0.049 (2)	−0.002 (2)	−0.0169 (19)	−0.0246 (19)
C19	0.091 (3)	0.067 (2)	0.061 (2)	0.014 (2)	−0.028 (2)	−0.036 (2)
C20	0.145 (4)	0.072 (3)	0.105 (3)	0.045 (3)	−0.066 (3)	−0.053 (2)
C21	0.136 (4)	0.169 (5)	0.117 (4)	0.050 (4)	−0.056 (3)	−0.100 (4)

### Geometric parameters (Å, °)

**Table:**

O1—C8	1.378 (3)	C6—H6A	0.9300
O1—C10	1.413 (3)	C7—C8	1.366 (4)
O2—C11	1.230 (3)	C7—H7	0.9300
O3—C15	1.369 (3)	C8—C9	1.413 (4)
O3—C19	1.437 (3)	C10—C11	1.497 (4)
O4—C16	1.366 (3)	C10—H10A	0.9700
O4—C20	1.416 (3)	C10—H10B	0.9700
O5—H21	0.8500	C12—C13	1.448 (4)
O5—H22	0.8499	C12—H12	0.9300
O6—C21	1.369 (4)	C13—C18	1.382 (4)
O6—H6	0.8200	C13—C14	1.401 (4)
N1—C1	1.315 (4)	C14—C15	1.366 (4)
N1—C9	1.359 (3)	C14—H14	0.9300
N2—C11	1.339 (3)	C15—C16	1.400 (4)
N2—N3	1.383 (3)	C16—C17	1.368 (4)
N2—H8	0.8600	C17—C18	1.397 (4)
N3—C12	1.271 (3)	C17—H17	0.9300
C1—C2	1.403 (5)	C18—H18	0.9300
C1—H1	0.9300	C19—H19A	0.9600
C2—C3	1.346 (4)	C19—H19B	0.9600
C2—H2	0.9300	C19—H19C	0.9600
C3—C4	1.403 (4)	C20—H20A	0.9600
C3—H3	0.9300	C20—H20B	0.9600
C4—C5	1.408 (4)	C20—H20C	0.9600
C4—C9	1.415 (4)	C21—H21A	0.9600
C5—C6	1.347 (4)	C21—H21B	0.9600
C5—H5	0.9300	C21—H21C	0.9600
C6—C7	1.407 (4)		
			
C8—O1—C10	115.6 (2)	O2—C11—N2	124.2 (3)
C15—O3—C19	116.5 (2)	O2—C11—C10	117.2 (3)
C16—O4—C20	117.6 (2)	N2—C11—C10	118.7 (3)
H21—O5—H22	105.8	N3—C12—C13	120.9 (3)
C21—O6—H6	109.5	N3—C12—H12	119.5
C1—N1—C9	116.9 (3)	C13—C12—H12	119.5
C11—N2—N3	117.4 (3)	C18—C13—C14	118.3 (3)
C11—N2—H8	121.3	C18—C13—C12	120.5 (3)
N3—N2—H8	121.3	C14—C13—C12	121.1 (3)
C12—N3—N2	116.4 (3)	C15—C14—C13	121.0 (3)
N1—C1—C2	124.2 (3)	C15—C14—H14	119.5
N1—C1—H1	117.9	C13—C14—H14	119.5
C2—C1—H1	117.9	C14—C15—O3	125.0 (3)
C3—C2—C1	119.1 (3)	C14—C15—C16	120.1 (3)
C3—C2—H2	120.4	O3—C15—C16	114.9 (3)
C1—C2—H2	120.4	O4—C16—C17	125.0 (3)
C2—C3—C4	119.6 (3)	O4—C16—C15	115.2 (3)
C2—C3—H3	120.2	C17—C16—C15	119.8 (3)
C4—C3—H3	120.2	C16—C17—C18	120.0 (3)
C3—C4—C5	123.1 (3)	C16—C17—H17	120.0
C3—C4—C9	117.3 (3)	C18—C17—H17	120.0
C5—C4—C9	119.6 (3)	C13—C18—C17	120.8 (3)
C6—C5—C4	120.3 (3)	C13—C18—H18	119.6
C6—C5—H5	119.9	C17—C18—H18	119.6
C4—C5—H5	119.9	O3—C19—H19A	109.5
C5—C6—C7	120.9 (3)	O3—C19—H19B	109.5
C5—C6—H6A	119.5	H19A—C19—H19B	109.5
C7—C6—H6A	119.5	O3—C19—H19C	109.5
C8—C7—C6	120.3 (3)	H19A—C19—H19C	109.5
C8—C7—H7	119.9	H19B—C19—H19C	109.5
C6—C7—H7	119.9	O4—C20—H20A	109.5
C7—C8—O1	124.2 (3)	O4—C20—H20B	109.5
C7—C8—C9	120.2 (3)	H20A—C20—H20B	109.5
O1—C8—C9	115.6 (3)	O4—C20—H20C	109.5
N1—C9—C8	118.5 (3)	H20A—C20—H20C	109.5
N1—C9—C4	122.9 (3)	H20B—C20—H20C	109.5
C8—C9—C4	118.6 (3)	O6—C21—H21A	109.5
O1—C10—C11	113.2 (3)	O6—C21—H21B	109.5
O1—C10—H10A	108.9	H21A—C21—H21B	109.5
C11—C10—H10A	108.9	O6—C21—H21C	109.5
O1—C10—H10B	108.9	H21A—C21—H21C	109.5
C11—C10—H10B	108.9	H21B—C21—H21C	109.5
H10A—C10—H10B	107.8		

### Hydrogen-bond geometry (Å, °)

**Table:**

*D*—H···*A*	*D*—H	H···*A*	*D*···*A*	*D*—H···*A*
N2—H8···O1	0.86	2.35	2.698 (3)	105
N2—H8···O5^i^	0.86	2.06	2.899 (3)	165
O6—H6···O2	0.82	1.98	2.756 (4)	156
O6—H6···N3	0.82	2.58	3.194 (4)	133
O5—H21···N1^ii^	0.85	2.00	2.834 (4)	168
O5—H22···O6^iii^	0.85	2.02	2.847 (4)	164

Symmetry codes: (i) *x*, *y*, *z*+1; (ii) *x*, *y*, *z*−1; (iii) −*x*+1, −*y*+1, −*z*+1.
